# The risk of new-onset type 2 diabetes and the influence of risk factors among men with prostate cancer in the Danish Diet, Cancer, and Health study

**DOI:** 10.1007/s11764-025-01776-7

**Published:** 2025-04-15

**Authors:** Jeppe Lyngbye Widding, Monika Barsøe, Gunn Ammitzbøll, Susanne Oksbjerg Dalton, Maja Halgren Olsen, Anne Katrine Graudal Levinsen, Klaus Brasso, Tinne Laurberg, Anne Tjønneland, Signe Benzon Larsen

**Affiliations:** 1 Cancer Survivorship, Danish Cancer Institute, Copenhagen, Denmark; 2 Urological Research Unit, Department of Urology, Copenhagen University Hospital – Rigshospitalet, Copenhagen, Denmark; 3 Department of Clinical Oncology & Palliative Care, Zealand University Hospital, Naestved, Denmark; 4 Department of Clinical Medicine, University of Copenhagen, Copenhagen, Denmark; 5 Steno Diabetes Center Aarhus, Aarhus University Hospital, Aarhus, Denmark; 6 Department of Pathology, Aarhus University Hospital, Aarhus, Denmark; 7 Diet, Cancer and Health, Danish Cancer Institute, Copenhagen, Denmark; 8 Department of Public Health, Section of Environmental Health, University of Copenhagen, Copenhagen, Denmark; 9 Section of Epidemiology, Department of Public Health, University of Copenhagen, Copenhagen, Denmark

**Keywords:** Cancer survivorship, Late effects, Nested case–control, Register study, Androgen deprivation therapy

## Abstract

**Purpose:**

To investigate the risk of new-onset type 2 diabetes mellitus (T2DM) in men with and without prostate cancer (PCa) and the influence of cardiometabolic risk factors. Furthermore, we examined the association between first-line androgen deprivation therapy (ADT) and the risk of T2DM in a subpopulation of men with PCa.

**Methods:**

In the Danish prospective Diet, Cancer, and Health cohort, we identified 2604 men with PCa and 9340 PCa-free men for comparison. We used Cox regression models to investigate the hazard ratios (HR) of T2DM from the date of diagnosis.

**Results:**

During 100,408 person-years at risk, 887 men were diagnosed with T2DM. Overall, no increased hazard of T2DM was observed in men with PCa compared to PCa-free men, but within the first year after PCa diagnosis, the HR of T2DM was 70% increased. In all men, the HRs for T2DM increased markedly with overweight/obesity, while comorbidity was associated with a pronounced increased HR in men with PCa. Within the PCa subpopulation, no association between ADT and T2DM was observed.

**Conclusion:**

Men with PCa have no overall increased risk of being diagnosed with T2DM compared to PCa-free men, despite an increased risk within the first year following cancer diagnosis, likely affected by increased surveillance.

**Implications for Cancer Survivors:**

Overall, survivors of PCa should not be more concerned about T2DM than PCa-free men; first-line treatment does not change the overall risk of T2DM in survivors of PCa. However, cardiometabolic risk factors require awareness to mitigate the risk of T2DM.

**Supplementary Information:**

The online version contains supplementary material available at https://doi.org/10.1007/s11764-025-01776-7.

## Introduction

Prostate cancer (PCa) is the most frequent cancer among Danish men, with a 5-year survival rate above 90% [1, 2]. Due to the favorable prognosis, the treatment of PCa often has a larger impact on health status and quality of life (QoL) than the disease itself [3]. Cardiometabolic diseases are among the most common late effects after PCa treatment and compromise both quality of life and survival. However, little is known about the interaction between PCa, PCa treatment, and development of cardiometabolic diseases [3, 4].

Type 2 diabetes mellitus (T2DM) is a common chronic disease associated with short- and long-term health consequences [5]. Diabetes and cancer share risk factors like age, overweight/obesity, smoking, alcohol consumption, physical inactivity, and morbidities; thus, the occurrence of both is expected to increase further in the future with growing longevity [5–7]. However, despite cancer and T2DM sharing risk factors, a link between PCa and T2DM has not yet been fully established [8–12]. Previous studies have observed that men with PCa overall have a similar or decreased risk of T2DM compared to the general population [8, 9]. However, studies in men with PCa receiving androgen deprivation therapy (ADT) have shown an increased risk of T2DM compared to men not receiving ADT [10–12]. Furthermore, cardiometabolic risk factors such as age, obesity, and lifestyle have not been appropriately accounted for in previous studies [8, 9]. These factors are likely to differ between men with and without PCa and may have an impact on the relationship between T2DM and PCa or PCa treatment.

Based on information from the prospective Danish Diet, Cancer, and Health (DCH) study, we had a unique opportunity to investigate the risk of new-onset T2DM in men with and without PCa and how this association may be altered by the cardiometabolic risk factors: age overweight/obesity, smoking, alcohol consumption, physical inactivity, and comorbidities. Moreover, we investigated the impact of first-line ADT on new-onset T2DM in men with PCa.

## Material and methods

### Study design

We conducted a nested case–control study following the STROBE Guidelines [13]. We combined information from the Danish prospective DCH cohort with information from nationwide registries, and electronic medical records, by use of the personal identification number assigned to all residents in Denmark [14].

### Study population

Between 1993 and 1997, 80,996 cancer-free men from Copenhagen and Aarhus aged 50–64 were invited to participate in the DCH cohort. In total, 26,944 men completed a questionnaire regarding their lifestyle and health. Trained healthcare workers performed anthropometric measurements and collected biological material. The DCH study was approved by the Regional Ethical Committees in Copenhagen and Aarhus (file number (KF) 11–037/01) and by the Danish Data Protection Agency. A detailed description of the DCH study is provided elsewhere [15].

**Fig. 1:**
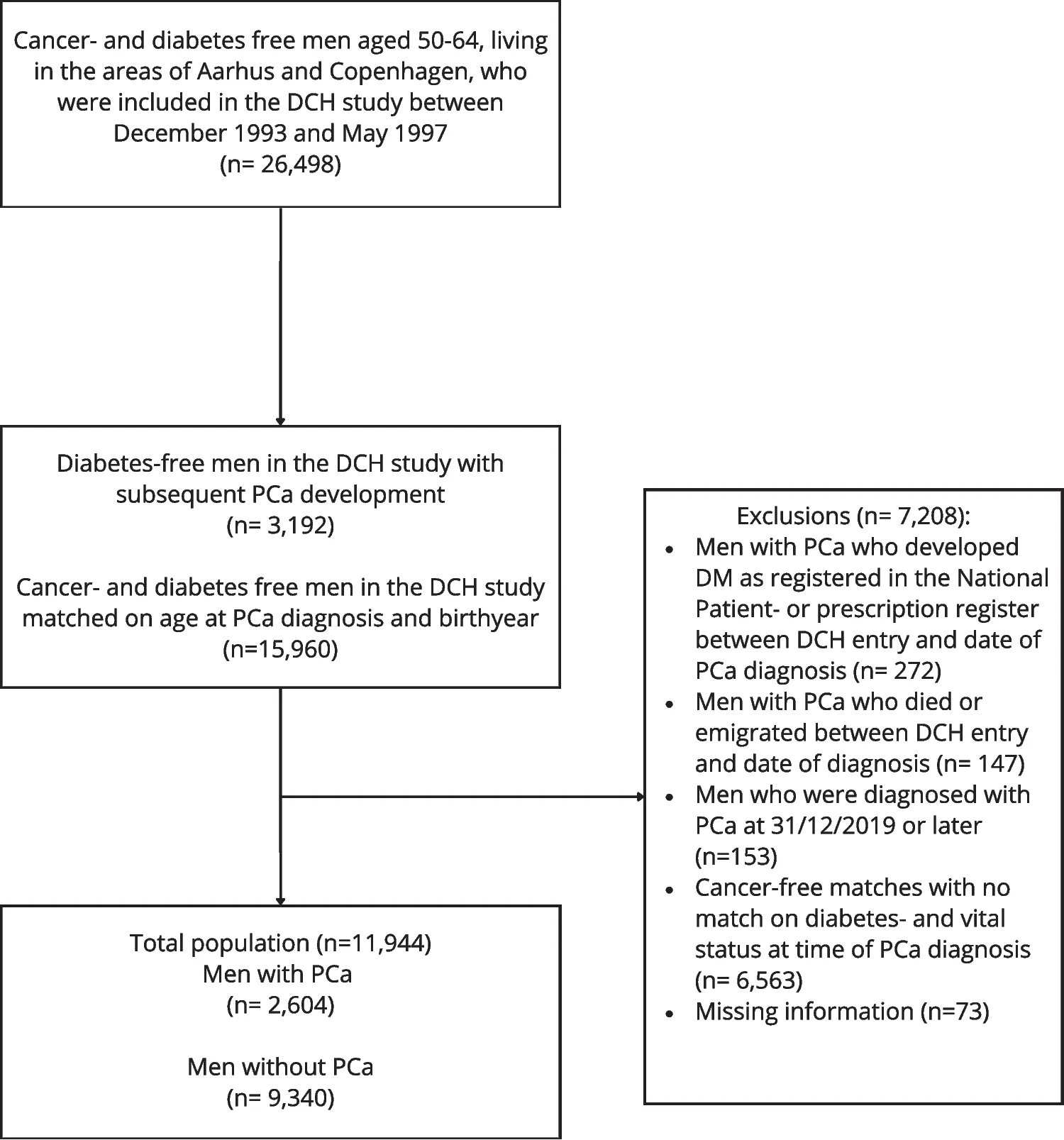
Flowchart of the population of the 2604 men from the Diet, Cancer, and Health study who were subsequently diagnosed with prostate cancer and the 9340 age-matched prostate cancer-free men. Abbreviations: DCH, Diet, Cancer, and Health; PCa, prostate cancer; DM, type 2 diabetes mellitus

In the DCH cohort, we identified 2604 men subsequently being registered in the Danish Cancer Registry with PCa between cohort enrollment and December 31, 2019 [16]. PCa cases were matched by age at the date of PCa diagnosis (index date) with PCa-free controls in the cohort, creating a comparison population of 9340 individuals. To be included, individuals had to be diabetes-free based on either self-reported diabetes status at DCH study enrollment or information from the National Patient Register and National Prescription Register and have complete information from the DCH cohort [14, 17, 18]. Figure 1 shows the flowchart of the study population. Additionally, we identified a subpopulation of 1683 PCa cases, diagnosed from 1995 to 2013, with complete information on first-line treatment from medical records (Supplementary Table 3).

### Outcome

The outcome of interest was new-onset T2DM. Information on the date of T2DM was obtained from the National Patient Register combined with information from the National Prescription Register, using a validated algorithm (Supplementary Table 1) [18, 19].

### Cardiometabolic risk factors

Age- and year of index date were included as categorical variables grouped in tertiles. Information on comorbidity three years before the index date was obtained from the National Patient Register and National Prescription Register to capture a stable view of the men’s disease burden. We computed this as a modified Charlson’s Comorbidity Index score by excluding PCa diagnoses, categorized into “0,” “1,” and “ ≥ 2” [20].

Information on anthropometric measurements, lifestyle, and education was obtained from enrollment in the DCH study. BMI was calculated and categorized into “healthy” (BMI < 25), “overweight” (BMI ≥ 25– < 30), and “obesity” (BMI ≥ 30). Waist circumference (WC) and waist-hip ratio (WHR) were categorized into tertiles. Physical activity level was described by the Metabolic Equivalents of Task (MET) score, which was calculated and categorized in tertiles [21]. Alcohol consumption was recorded as units of alcohol/week and categorized into tertiles. Smoking status was categorized into “current,” “former,” and “never”. Education was recorded as length of schooling and categorized into “short” (≤ 7 years), “medium” (8–10 years), and “long” (> 10 years).

### Androgen deprivation therapy

Information regarding first-line treatment was obtained from medical records at the time of PCa diagnosis up to the end of 2013. First-line treatment was categorized as “ADT” (androgen deprivation therapy), “conservative treatment” (active surveillance or watchful waiting), and “curative treatment” (radical prostatectomy or radiation therapy). We did not have information on the treatment of men diagnosed after 2013, and we excluded men who were not assigned to any specific treatment.

### Statistical analyses

Descriptive characteristics were stratified by men with PCa and the age-matched comparison population of PCa-free men. The cumulative incidence of T2DM was plotted with death as a competing risk; additionally, we plotted the cumulative incidence of death.

We investigated the impact of having a PCa diagnosis on being diagnosed with T2DM with univariate and multivariate Cox regression models. The multivariate models were adjusted for factors hypothesized to confound the association, including age, cardiometabolic risk factors, education, and the index year (Supplementary Fig. 1). We used years since the index date as the underlying timescale and death as a competing risk in all models. The study participants were followed from the index date until the date of T2DM, emigration, death, or end of follow-up (December 31, 2019), whichever occurred first.

First, we compared the hazards of T2DM among men with PCa and without PCa and investigated interactions between PCa and confounding variables. Additionally, we assessed the influence of time after the index date on the observed associations in separate models according to time since the index date (0–1 year, > 1–5 years, > 5–10 years, and > 10 years). Secondly, we estimated the association between cardiometabolic risk factors and T2DM in models separately in men with and without PCa. Finally, we restricted the analyses to 1683 men who were diagnosed with PCa through 2013 with information on first-line treatment, aiming to investigate the impact of first-line ADT treatment on the risk of being diagnosed with T2DM.

Estimates were expressed as crude or adjusted hazard ratios (HRs/aHRs) with 95% confidence intervals (95% CIs). We explored the Cox proportional hazards assumption in all models by inspecting the *p*-values of statistical assumption tests and Schoenfeld’s residual plots of all exposure and covariate variables. We entered education as a stratifying variable in all models due to the violation of the model assumption of proportional hazards. The level of statistical significance was set to 5%. Analyses were performed using R statistical Software version 4.3.3 [22].

## Results

### Study population

The mean age at the time of PCa diagnosis was 71 years (SD 5.8) (Table 1). Men with PCa had longer education, were slightly healthier in terms of anthropometrics and physical activity, had lower levels of severe comorbidity, and fewer were current smokers than PCa-free men.

**Table 1:** Characteristics of 2604 men diagnosed with prostate cancer from 1995 to 2019 and the 9340 age-matched men without prostate cancer from the Diet, Cancer, and Health study

	Men with prostate cancer, *n* (%)	Men without prostate cancer, *n* (%)
**Total numbers**	2604 (21.8)	9340 (78.2)
**Median follow-up time, years (IQR)**	6.1 (2.9, 10.4)	8.5 (4.5, 12.4)
**Mean age at diagnosis/index date, years (SD)**	71 (5.8)	70 (5.7)
**Year of diagnosis/index**		
1995–2007	950 (36.5)	3896 (41.7)
2008–2011	690 (26.5)	2490 (26.7)
2012–2019	964 (37.0)	2954 (31.6)
**Education**		
Long	649 (24.9)	2153 (23.1)
Medium	1,079 (41.4)	3892 (41.7)
Short	876 (33.6)	3295 (35.3)
**Charlson’s Comorbidity Index Score up to diagnosis/index date**		
0	2213 (85.0)	7817 (83.7)
1	274 (10.5)	1008 (10.8)
≥ 2	117 (4.5)	515 (5.5)
***Lifestyle-related factors at DCH study enrollment***		
**MET score in kcal/kg/h**		
≤ 42	871 (33.4)	3183 (34.1)
> 42–72	890 (34.2)	3118 (33.4)
> 72	843 (32.4)	3039 (32.5)
**Alcohol intake per week in units**		
≤ 7	1006 (38.6)	3538 (37.9)
> 7–14	764 (29.3)	2800 (30.0)
> 14	834 (32.0)	3002 (32.1)
**Smoking history**		
Never	780 (30.0)	2535 (27.1)
Former	948 (36.4)	3389 (36.3)
Current	876 (33.6)	3416 (36.6)
***Anthropometric at DCH study enrollment***		
**Body mass index**		
Healthy (BMI < 25)	975 (37.4)	3406 (36.5)
Overweight (BMI 25– < 30)	1349 (51.8)	4784 (51.2)
Obesity (BMI ≥ 30)	280 (10.8)	1150 (12.3)
**Waist circumference**		
≤ 91	938 (36.0)	3326 (35.6)
> 91–98	858 (32.9)	2938 (31.5)
> 98	808 (31.0)	3076 (32.9)
**Waist-hip ratio**		
≤ 0.93	1092 (41.9)	3729 (39.9)
> 0.93–0.97	716 (27.5)	2743 (29.4)
> 0.97	796 (30.6)	2868 (30.7)

*n* number, *SD* standard deviation, *IQR* interquartile range, *MET* metabolic equivalents of task, *DCH* Diet, Cancer, and Health study.

^a^Censored due to emigration, or end of follow-up (December 31, 2019).

### The cumulative incidence of type 2 diabetes mellitus and death

**Fig. 2:**
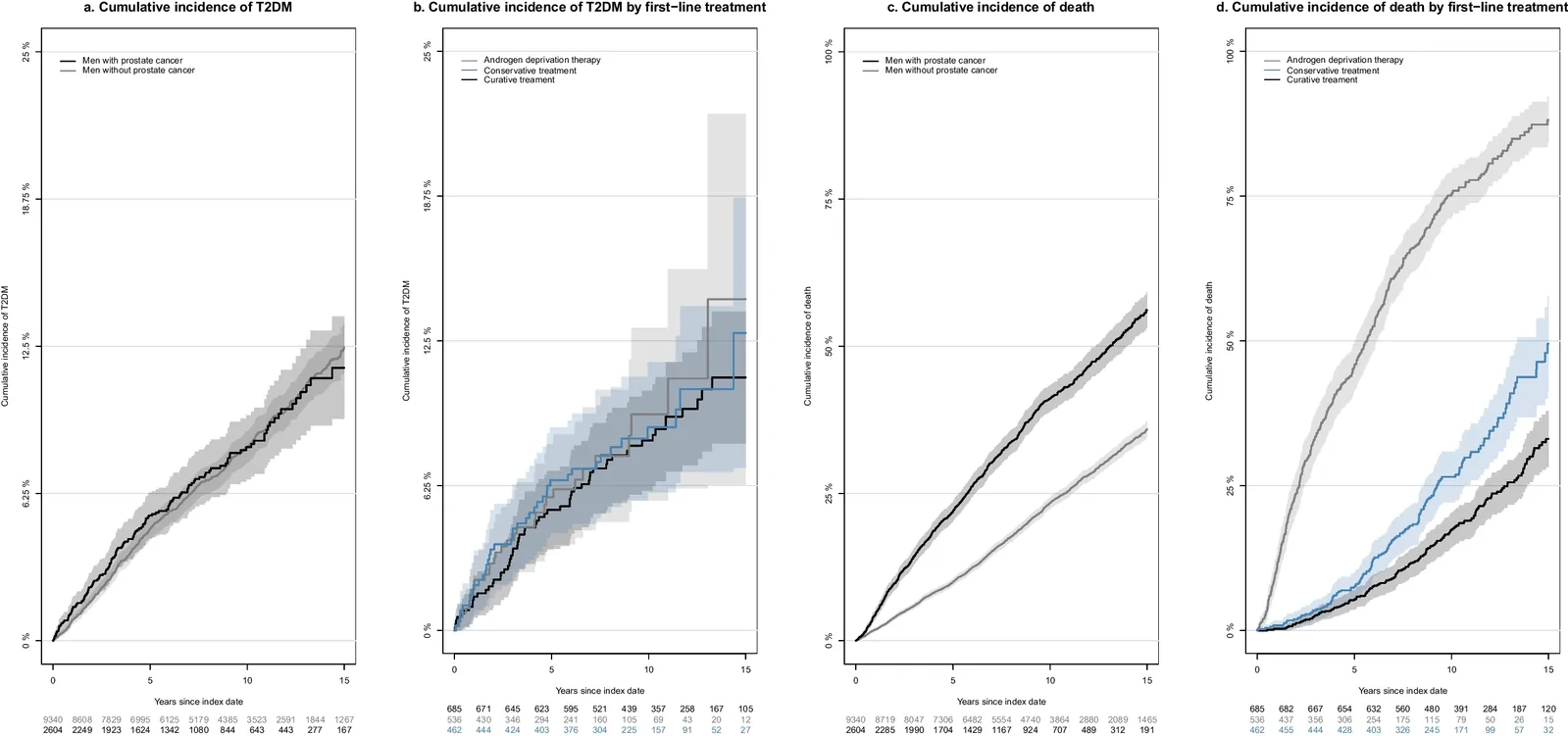
The cumulative incidence of type 2 diabetes mellitus and death among the 2,604 men diagnosed with prostate cancer from 1995 through 2019 and 9,340 age-matched men without prostate cancer in the Danish Diet, Cancer, and Health study, and the cumulative incidence of type 2 diabetes mellitus and death stratified by first-line treatment in the subpopulation of men with prostate cancer diagnosed from 1995 to 2013. **a** Cumulative incidence plot of new-onset type 2 diabetes among men with and without prostate cancer. **b** Cumulative incidence plot of new-onset type 2 diabetes stratified by first-line treatment in the subpopulation of men with prostate cancer. **c** Cumulative incidence plot of death among men with and without prostate cancer. **d** Cumulative incidence plot of death stratified by first-line treatment in the subpopulation of men with prostate cancer. Please note the varying percentages on the *y*-axis. Numbers below the bottom x legend are the at-risk individuals at each time point. Conservative treatment consisted of active surveillance or watchful waiting. Curative treatment consisted of radical prostatectomy or radiotherapy. Abbreviations: T2DM, type 2 diabetes mellitus

The cumulative incidence of T2DM was similar for men with and without PCa (Fig. 2). The cumulative incidence was 5%, 7%, and 10% after 5, 10, and 15 years, respectively. In the subpopulation, there was no differences in the cumulative incidence of T2DM according to first-line treatment during the 15 years of follow-up. However, men with PCa had a higher cumulative incidence of death than PCa-free men, and men with PCa treated with ADT had an increased cumulative incidence of death compared to men who did not receive ADT treatment.

### The hazard of type 2 diabetes mellitus

In a total of 100,408 person-years at risk, there were 887 incident diagnoses of T2DM (Table 2). Men with PCa had fewer person-years at risk (person-years at risk 18,118) and fewer incident T2DM diagnoses (*n* = 166) than men without PCa. Overall, the hazards of T2DM were similar for men with and without PCa, in both the univariate (HR 1.0, 95% CI 0.9–1.2) and multivariate model (aHR 1.1, 95% CI 0.9–1.3) (Table 2). However, during the first year after the index date, the HR of T2DM was 70% increased among men with PCa (HR 1.6, 95% CI 1.1–2.3) (aHR 1.7, 95% CI 1.1–2.5), while there was no increased HR after > 1–5 years, > 5–10 years, and > 10 years of follow-up (Table 2).

**Table 2:** The hazard ratios of type 2 diabetes mellitus among men with prostate cancer and age-matched men without prostate cancer in the Danish Diet, Cancer, and Health study

	*N* of men (%)	*N* of events (%)	Person-years at risk	Crude HR (CI 95%)	aHR (CI 95%) ^a^
**Total follow-up after index date**					
Men with PCa	2604 (22)	166 (6)	18,118	1.0 (0.9–1.2)	1.1 (0.9–1.3)
Men without PCa	9340 (78)	721 (8)	82,290	Reference	Reference
**0–1 year after index date**					
Men with PCa	2620 (22)	34 (1)	2503	1.6 (1.1–2.3)	1.7 (1.1–2.5)
Men without PCa	9340 (78)	79 (1)	9116	Reference	Reference
** > 1–5 years after index date**					
Men with PCa	2384 (21)	79 (3)	7714	1.0 (0.8–1.3)	1.1 (0.9–1.4)
Men without PCa	8881 (79)	310 (4)	31,197	Reference	Reference
** > 5–10 years after index date**					
Men with PCa	1533 (19)	34 (2)	5471	0.8 (0.6–1.2)	0.9 (0.6–1.3)
Men without PCa	6716 (81)	195 (3)	26,231	Reference	Reference
** > 10 years after index date**					
Men with PCa	721 (16)	19 (3)	2406	0.9 (0.5–1.4)	1.0 (0.6–1.6)
Men without PCa	3852 (84)	137 (6)	15,705	Reference	Reference

There were no significant interactions between PCa and any of the covariates (not shown).

*N* number, *PCa* prostate cancer, *HR* hazard ratio, *aHR* adjusted hazard ratio, *CI* confidence interval.

^a^Adjusted for education, Charlson’s Comorbidity Index Score, BMI, smoking, alcohol, metabolic equivalents of task score, age at index date, and year of index.

### Cardiometabolic risk factors in men with and without prostate cancer

**Fig. 3:**
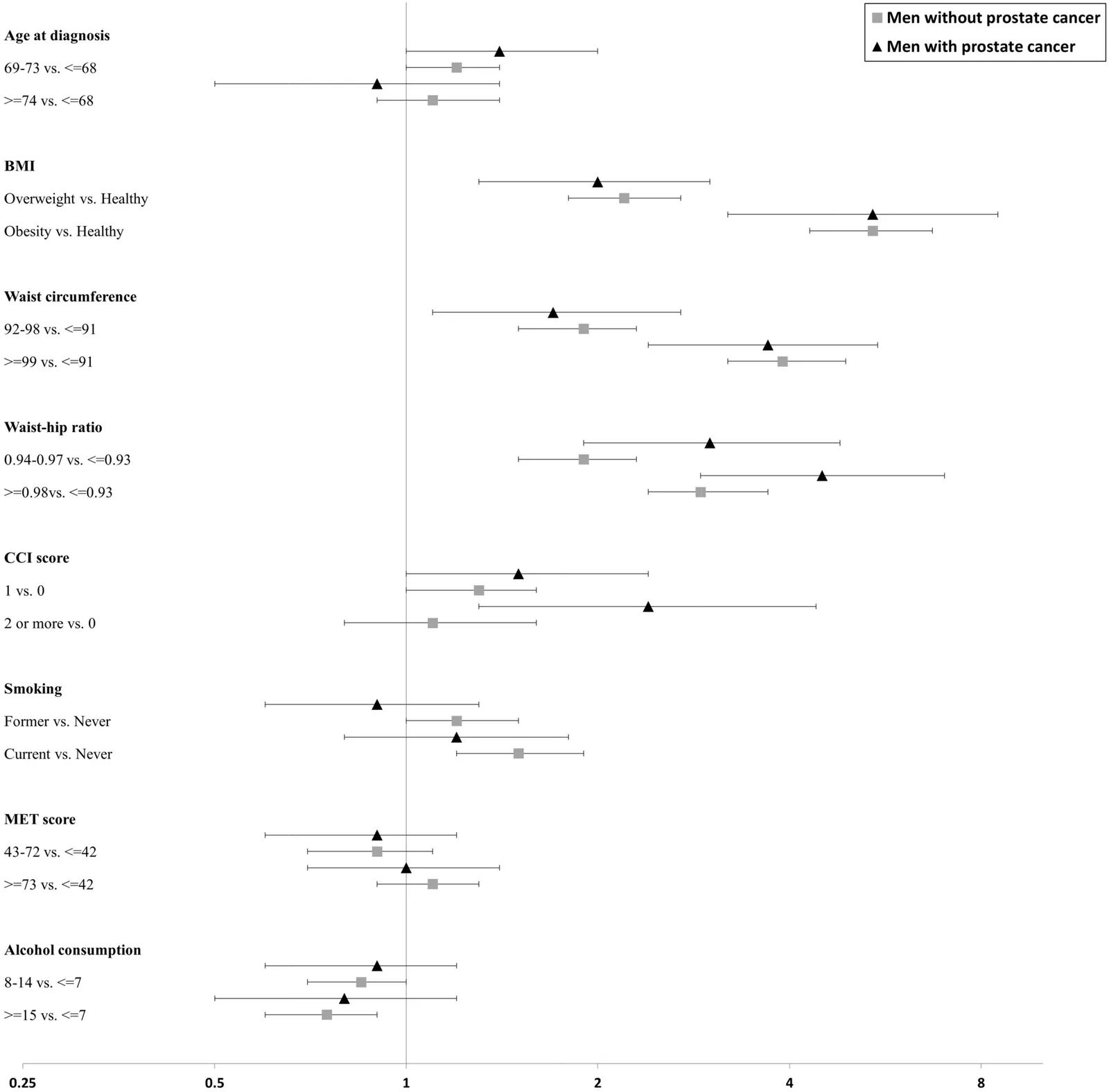
Adjusted hazard ratios with 95% confidence intervals of type 2 diabetes mellitus in cardiometabolic risk factors stratified by men with or without prostate cancer. Corresponding estimates are provided in Supplementary Table 4. Abbreviations: CCI, Charlson’s Comorbidity Index; MET, metabolic equivalents of task

In men with and without PCa, those aged 69–73 years had an increased hazard of T2DM compared to men aged 68 years or younger, while there was no increased hazard among men aged 74 years or older (Fig. 3). Increased BMI or WC was associated with an increased hazard of T2DM compared to men with healthy BMI, or a WC of 91 or lower, respectively, while there was no association with physical activity and alcohol consumption. In all men, increased WHR was associated with an increased hazard of T2DM, but the aHRs were more pronounced in men with PCa. Exclusively in men with PCa, having comorbidity was associated with an increased hazard of T2DM compared to no comorbidity, while smoking increased the hazard of T2DM in men without PCa solely (Fig. 3).

### Impact of androgen deprivation therapy

**Fig. 4:**
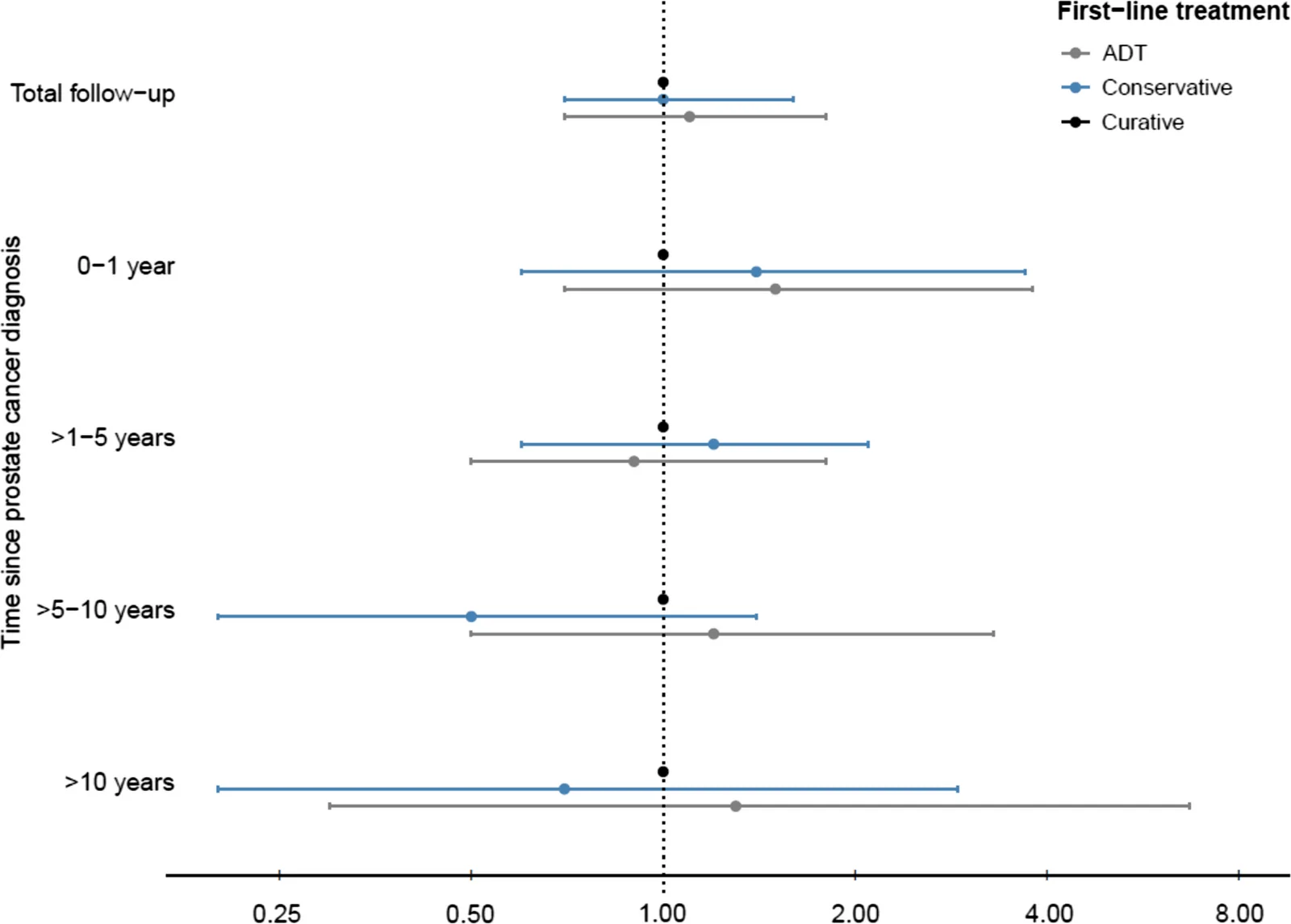
The adjusted hazard ratios with 95% confidence intervals of type 2 diabetes mellitus associated with first-line treatment and the influence of time since diagnosis in the subpopulation of 1,683 men diagnosed with prostate cancer from 1995 to 2013. Conservative treatment included active surveillance or watchful waiting; curative treatment included radical prostatectomy or radiation therapy. Models were adjusted for education, smoking, BMI, Charlson’s Comorbidity Index Score, alcohol, metabolic equivalents of task score, age at index, and year of index. Corresponding estimates are provided in supplementary Table 5, and characteristics of the subpopulation of men with PCa are provided in supplementary Table 4. Abbreviations: ADT, androgen deprivation therapy

In the subpopulation of men with PCa, those treated with ADT as first-line treatment did not have an increased hazard of T2DM compared to those treated curatively in the total follow-up period (HR 1.1, 95% CI 0.7–1.7) (aHR 1.1, 95% CI 0.7–1.8) (Fig. 4). We found no increased hazard of being diagnosed with T2DM during the first year (aHR 1.5, 95% CI 0.6–3.7), or in the remaining follow-up (> 1–5 years, > 5–10 years, and > 10 years of follow-up) (Fig. 4).

## Discussion

In this large prospective cohort study, we found no overall increased risk of being diagnosed with T2DM in men with PCa; however, the risk was increased within the first year after PCa diagnosis, compared to men without PCa. Overweight/obesity (i.e., increased BMI, WC, and WHR) was the risk factor with the strongest association with T2DM among all men, and despite only minor differences in the influence of cardiometabolic risk factors, comorbidity indicated an increased risk for T2DM among men with PCa. Lastly, in the subpopulation of men with PCa, first-line ADT treatment was not associated with an increased risk of T2DM when compared to men treated with curative treatment.

We found that PCa was not associated with an increased hazard of T2DM regardless of age, education, BMI, comorbidity, lifestyle, and year of diagnosis (Table 2). A Korean cohort study found no statistical difference in the risk of T2DM (HR 0.81, 95% CI 0.55–1.20) among men with PCa compared to PCa-free men after adjustment for age, metabolic factors, cardiometabolic risk factors (BMI, smoking, alcohol intake, and comorbidities) [9]. The authors excluded diabetes cases that occurred during the first 31 days after cancer diagnosis, making their results less likely to be influenced by surveillance bias compared to our study. In contrast, a previous Danish cohort study found a reduced risk of T2DM among men with PCa compared to PCa-free men (HR 0.86, 95% CI 0.76–0.96), using laboratory samples from general practitioners as a diabetes identifier, which may explain their contradictory findings [8]. A previous Danish register-based study observed an increased proportion of new prescriptions of antidiabetics synchronous with PCa diagnosis but no differences in proportion of prescriptions were observed between men with and without PCa in the years after diagnosis [23]. These findings indicate that undiagnosed T2DM is more likely to be detected synchronous with the diagnostic investigation for PCa, where contact with the healthcare system increases and therefore leads to a small effect of surveillance bias in the years following the PCa diagnosis.

The biological mechanism between cancer and T2DM is complex and not sufficiently known [24]. Cancer cachexia has been suggested as an underlying mechanism for the development of T2DM among cancer patients [8]. As men with PCa are often diagnosed at an early stage, most will remain unaffected by cachexia, which could contribute to the absence of an increased risk among men with PCa. This is supported by other studies that find an increased risk of T2DM in men with cancer types where cachexia is a common occurrence [8, 9]. In summary, the current study and the most recent literature indicate that men with PCa do not have an increased risk of new-onset diabetes compared to PCa-free men.

The influence of time since PCa diagnosis on the risk of T2DM has only been evaluated among men with PCa by the duration of ADT. Crawley et al. found a 50% increased risk in men with PCa on ADT after 0.5–1 year from diagnosis, compared to ADT-free men [25], and Drevinskaite et al. found a 40% increased risk within the first year after diagnosis among men with PCa on ADT, compared to ADT-free men with PCa [26]. In all men with PCa, we found a 70% increased risk in the first year after PCa diagnosis, compared to men without PCa (Table 2). Although cancer treatment could contribute to the development of T2DM within the first year after cancer diagnosis, we interpret our finding as surveillance bias. It is well known that cancer patients have more frequent contact with the healthcare system and medical examinations after cancer diagnosis, especially in the initial period, compared to the general population. This is supported by the fact that we found no increased risk in the following periods when the surveillance may naturally have decreased in intensity.

The influence of cardiometabolic risk factors on the risk of T2DM may be more prominent among men with PCa than in men without PCa, due to a potential interaction with PCa and PCa treatment. However, our results did not support an interaction. Obesity-related anthropometrics were the strongest predictors of new-onset T2DM among all men in this study, while overall, we only observed minor variations in how risk factors influenced the risk of T2DM in men with and without PCa (Fig. 3). This may be due to differences in the level or type of comorbidity, interaction of these factors with PCa or PCa treatment, or surveillance bias. Despite overlapping CIs, our results indicated that having comorbidity or increased WHR had a slightly larger influence on the risk of diabetes among men with PCa. The latter finding may be explained due to a large clustering of risk factors and diseases that increase the likelihood of T2DM among men with PCa. The stronger association between WHR and T2DM among men with PCa may be attributed to the visceral fat level [27], known to be linked to insulin resistance [28], which has been suggested to be more pronounced among patients with cancer in general [29]. More studies need to confirm these findings in men with PCa.

In the first study of Danish men with PCa, about ADT as a risk factor for T2DM, we did not find evidence of an association (Fig. 4). However, the association has previously been evaluated in several systematic reviews, and the current evidence supports that patients who receive ADT have an approximately 30% increased relative risk compared to patients who do not receive ADT [10–12]. ADT decreases testosterone levels to castration levels [30]. This effect on testosterone levels may increase cardiometabolic risk factors which could lead to diabetes or exacerbate pre-diabetic conditions [31]. Furthermore, as most men who receive ADT are diagnosed with non-localized disease, cancer cachexia may potentially mediate an increased risk of T2DM compared to patients receiving other treatments (Supplementary Table 3). However, our contrasting finding may be explained by the fact that we only had information on the assigned first-line treatment. Many men with PCa are assigned other treatments later, because of recurrence or progression, and some men in Denmark who are assigned to curative intent are treated with first-line radiation therapy and additional intermittent ADT for three years [32]. This could potentially have added more ADT users to the group of men treated with curative intent in this study.

### Strengths and limitations

The prospective design with data linkage to national registries secured a long and nearly complete follow-up of the participants. We used a validated algorithm for identifying the outcome of interest (new-onset T2DM) [19], and information on the exposures was collected independently of the outcome of this study through validated questionaries and measurements performed by trained healthcare workers in the DCH cohort. Regardless of the high quality of the information on the cardiometabolic risk factors measured at DCH entry, it is a limitation that these measurements were performed before PCa diagnosis (range 1–25 years). The men’s exposure status in these cardiometabolic risk factors may have been misclassified due to changes in weight and lifestyle in the period between DCH entry and PCa diagnosis. Further, we only had information on first-line treatment and clinical cancer characteristics up to the end of 2013, which only made it possible to investigate the association between ADT and the risk of T2DM in a subpopulation of men with PCa, influencing the generalizability of these findings. More, it has previously been shown that male participants of the DCH study generally had a higher educational level, were more likely to be working or married, and had a lower mortality compared to non-participants (15, 33). Self-selection into the DCH study might thus influence the generalizability of the findings from the present study but is unlikely to affect the strength of the relative associations.

## Conclusion

Despite an increased risk within the first year following cancer diagnosis, men with PCa have no overall increased risk of being diagnosed with T2DM compared to PCa-free men. Increased surveillance within the first year after diagnosis might explain parts of the increased risk. Overweight and obesity were the strongest cardiometabolic predictors of T2DM in all men, and although comorbidity was a pronounced risk factor in men with PCa, there were only small variations in the overall influence of cardiometabolic risk factors regardless of cancer status. Remarkably, our study revealed that first-line ADT was not associated with an increased risk of T2DM in the subpopulation of men with PCa.

These findings provide insight that may be of relevance in clinical practice. Clinicians should monitor diabetes and risk factors, regardless of the presence of PCa diagnosis or follow-up timing. Notably, concerns about new-onset T2DM should not influence shared decision-making regarding first-line treatment in men with PCa.

## Implications for cancer survivors

Overall, survivors of PCa should not be more concerned about T2DM than PCa-free men although frequently contact with the healthcare system may increase the risk of being diagnosed shortly after cancer diagnosis. First-line treatment does not modify the risk of T2DM in survivors of PCa. However, cardiometabolic risk factors require awareness to mitigate the risk of T2DM.

## Supplementary Information

Below is the link to the electronic supplementary material.

Supplementary file1 (DOCX 103 KB)

## Data Availability

Access to data can be obtained by request to the corresponding authors, the Diet, Cancer, and Health cohort, and Danish Data protection authorities. Additional data has been provided in supplemental files.
