# Supplementary material for: The risk of new-onset type 2 diabetes and the influence of risk factors among men with prostate cancer in the Danish Diet, Cancer, and Health study
Source: J Cancer Surviv. 2025 Apr 15;20(Suppl 1):153–63. doi: 10.1007/s11764-025-01776-7 (PMC13676483; doi:10.1007/s11764-025-01776-7)
Supplement: Supplementary file 1 [file 11764_2025_1776_MOESM1_ESM.docx]

**Supplementary materials**

**Figure 1.** Illustration of the proposed relationship between prostate cancer, confounding factors, mediators, and new-onset type 2 diabetes mellitus.

Diagnostic information on age and calendar year of prostate cancer diagnosis was collected through the Danish Cancer Registry. Pre-diagnostic information was collected at DCH study enrollment by validated questionaries or measurements performed by healthcare staff, except for comorbidity history, which was collected through the National Patient- and Prescription Registers. These factors were considered to potentially confound the relationship between prostate cancer and new-onset type 2 diabetes mellitus. Information from medical records was collected around the time of prostate cancer diagnosis and these factors were considered to potentially mediate the relationship between prostate cancer and new-onset type 2 diabetes mellitus.

**Supplementary Table 1.** The algorithm for identifying type 1 and 2 diabetes mellitus.

|  | **Type 2 diabetes mellitus** | **Type 1 diabetes mellitus** |
| --- | --- | --- |
| **Inclusion criteria** | - A purchase of non-insulin glucose-lowering drugs (ATC group: A10B)  Or  - A hospital event for type-specific diabetes diagnosis: Type 2 diabetes (ICD-10 code: E11 and subcategories). | - A purchase of insulin (ATC group: A10A)  Or  a hospital event, with the last type-specific diabetes diagnosis: Type 1 diabetes (ICD-10 code: E10 and subcategories). |
| **Exclusion criteria** | - Only one inclusion event (purchase of non-insulin glucose-lowering drug or type 2 diabetes diagnosis)  - The last purchase of a non-insulin glucose-lowering drug purchases or last hospital event for type 2 diabetes, was more than 10 years before the index date. | - The last purchase of insulin was more than 10 years before the index date.  Or  - Only one insulin purchase and no type 1 diabetes diagnoses.  Or  - Classified as type 2 diabetes according to the first column. |

Abbreviations: ATC= Anatomical Therapeutic Chemical Classification System, ICD= International Classification of Diseases.

**Supplementary Table 2.** The algorithm used for the staging of prostate cancer.

|  | **Localized** | **Non-localized** |
| --- | --- | --- |
|  | C_udbred = 5 ^a^ | c_udbred= 6 or 7 ^b^ |
| or | T1-2 | T3-T4 |
| or | N0 | N1 |
| or | M0 | M1 |

Abbreviations: T= Tumor, N= Node, M= Metastases. ^a^ No tumor spread according to previous cancer classification system. ^b^ Tumor spread according to previous cancer classification system.

**Supplementary Table 3.** Characteristics of the sub-population of 1,683 men with prostate cancer diagnosed from 1995 through 2013 stratified by first-line treatment.

|  | ADT, n (%) | Conservative, n (%) | Curatively, n (%) |
| --- | --- | --- | --- |
| Total numbers | 536 (32.0) | 462 (28.0) | 685 (40.0) |
| T stage |  |  |  |
| 1 | 139 (30.0) | 321 (76.2) | 256 (42.2) |
| 2 | 67 (14.5) | 74 (17.6) | 220 (36.2) |
| 3 or 4^a^ | 226 (48.8) | 19 (4.5) | * |
| x | 31 (6.7) | 7 (1.7) | * |
| Missing (%) | 11.4 | | |
| N stage |  |  |  |
| 0 | 31 (6.3) | 45 (10.4) | 311 (48.0) |
| 1 | 117 (23.6) | 6 (1.4) | 10 (1.5) |
| x | 347 (70.1) | 381 (88.2) | 327 (50.5) |
| Missing (%) | 6.4 | | |
| M stage |  |  |  |
| 0 | 189 (37.0) | 160 (36.9) | 458 (70.1) |
| 1 | 227 (44.4) | 6 (1.4) | 10 (1.5) |
| x | 95 (18.6) | 268 (61.8) | 185 (28.3) |
| Missing (%) | 5.1 | | |
| Cancer stage |  |  |  |
| Localized | 172 (35.0) | 400 (94.1) | 503 (78.3) |
| Non-localized | 320 (65) | 25 (5.9) | 139 (21.7) |
| Missing (%) | 7.4 | | |
| Gleason score |  |  |  |
| ≤6 | 28 (5.9) | 265 (64.2) | 237 (37.2) |
| 7 | 157 (33.3) | 125 (30.3) | 260 (40.6) |
| ≥8 | 287 (60.8) | 23 (5.6) | 142 (22.2) |
| Missing (%) | 10.0 | | |
| PSA level |  |  |  |
| ≤10 | 44 (8.5) | 289 (64.1) | 353 (52.8) |
| 10-20 | 75 (14.6) | 113 (25.1) | 199 (29.8) |
| >20 | 396 (76.9) | 49 (10.9) | 116 (17.4) |
| Missing (%) | 2.9 | | |

Abbreviations: N= number, ADT= Androgen deprivation therapy, T= Tumor, N= Node, M= Metastases, PSA= Prostate-specific antigen. *Not reported due to low numbers / data cannot be included in the table, as they present information on less than five individuals

**Supplementary Table 4.** Hazard ratios for factors associated with risk of new-onset type 2 diabetes mellitus in men with prostate cancer and the age-matched men without prostate cancer.

|  | Men with prostate cancer | | | | Men without prostate cancer | | | |
| --- | --- | --- | --- | --- | --- | --- | --- | --- |
|  | N of men (%) | N of events  (%) | Crude HR (CI 95%) | aHR  (CI 95%) | N of men (%) | N of events (%) | Crude HR  (CI 95%) | aHR  (CI 95%) |
| Age at index |  |  |  |  |  |  |  |  |
| T1 (≤68) | 814 (31) | 68 (8) | Reference | Reference ^a^ | 3,343 (36) | 355 (11) | Reference | Reference ^b^ |
| T2 (69-73) | 848 (33) | 70 (8) | 1.2 (0.9-1.8) | 1.4 (1.0-2.0) ^a^ | 3,149 (34) | 247 (8) | 1.1 (0.9-1.3) | 1.2 (1.0-1.4) ^a^ |
| T3 (≥74) | 942 (36) | 28 (3) | 0.7 (0.4-1.0) | 0.9 (0.5-1.4) ^a^ | 2,848 (30) | 119 (4) | 0.9 (0.7-1.1) | 1.1 (0.9-1.4) ^a^ |
| BMI |  |  |  |  |  |  |  |  |
| Healthy | 975 (37) | 33 (3) | Reference | Reference ^b^ | 3,406 (36) | 128 (4) | Reference | Reference ^b^ |
| Overweight | 1,349 (52) | 85 (12) | 1.9 (1.3-2.9) | 2.0 (1.3-3.0) ^b^ | 4,784 (51) | 379 (8) | 2.2 (1.8-2.6) | 2.2 (1.8-2.7) ^b^ |
| Obesity | 280 (11) | 48 (17) | 5.6 (3.6-8.7) | 5.4 (3.4-8.5) ^b^ | 1,150 (12) | 214 (19) | 5.4 (4.4-6.7) | 5.4 (4.3-6.7) ^b^ |
| WC |  |  |  |  |  |  |  |  |
| T1 (≤91) | 938 (36) | 31 (3) | Reference | Reference ^b^ | 3,326 (36) | 119 (4) | Reference | Reference ^b^ |
| T2 (92-98) | 858 (33) | 46 (5) | 1.7 (1.1-2.6) | 1.7 (1.1-2.7) ^b^ | 2,938 (31) | 192 (7) | 1.9 (1.5-2.3) | 1.9 (1.5-2.3) ^b^ |
| T3 (≥99) | 808 (31) | 89 (11) | 3.7 (2.4-5.5) | 3.7 (2.4-5.5) ^b^ | 3,076 (33) | 410 (13) | 4.0 (3.2-4.9) | 3.9 (3.2-4.9) ^b^ |
| WHR |  |  |  |  |  |  |  |  |
| T1 (≤0.93) | 1,092 (42) | 27 (2) | Reference | Reference ^b^ | 3,729 (40) | 156 (4) | Reference | Reference |
| T2 (0.94-0.97) | 716 (27) | 52 (7) | 3.0 (1.9-4.7) | 3.0 (1.9-4.8) ^b^ | 2,743 (29) | 216 (8) | 1.9 (1.6-2.4) | 1.9 (1.5-2.3) ^b^ |
| T3 (≥0.98) | 796 (31) | 87 (11) | 4.6 (3.0-7.1) | 4.5 (2.9-7.0) ^b^ | 2,868 (31) | 349 (12) | 3.1 (2.5-3.7) | 2.9 (2.4-3.7) ^b^ |
| CCI score |  |  |  |  |  |  |  |  |
| 0 | 2,213 (85) | 132 (6) | Reference | Reference ^c^ | 7,817 (84) | 609 (8) | Reference | Reference ^c^ |
| 1 | 274 (11) | 22 (8) | 1.5 (1.0-2.4) | 1.5 (1.0-2.4) ^c^ | 1,008 (11) | 80 (8) | 1.3 (1.1-1.7) | 1.3 (1.0-1.6) ^c^ |
| 2+ | 117 (5) | 12 (10) | 2.5 (1.4-4.4) | 2.4 (1.3-4.4) ^c^ | 515 (5) | 32 (6) | 1.4 (1.0-1.9) | 1.1 (0.8-1.6) ^c^ |
| Smoking |  |  |  |  |  |  |  |  |
| Never | 780 (30) | 49 (6) | Reference | Reference ^d^ | 2,535 (27) | 159 (6) | Reference | Reference ^d^ |
| Former | 948 (36) | 58 (6) | 1.0 (0.7-1.4) | 0.9 (0.6-1.3) ^d^ | 3,389 (36) | 284 (8) | 1.3 (1.1-1.6) | 1.2 (1.0-1.5) ^d^ |
| Current | 876 (34) | 59 (7) | 1.2 (0.8-1.8) | 1.2 (0.8-1.8) ^d^ | 3,416 (37) | 278 (8) | 1.4 (1.1-1.7) | 1.5 (1.2-1.9) ^d^ |
| Met score |  |  |  |  |  |  |  |  |
| T1 (≤42) | 871 (33) | 62 (7) | Reference | Reference ^f^ | 3,183 (34) | 257 (8) | Reference | Reference ^f^ |
| T2 (43-72) | 890 (34) | 48 (5) | 0.8 (0.5-1.1) | 0.8 (0.6-1.2) ^f^ | 3,118 (33) | 213 (7) | 0.7 (0.7-1.0) | 0.9 (0.7-1.1) ^f^ |
| T3 (≥73) | 843 (32) | 56 (7) | 0.9 (0.7-1.4) | 1.0 (0.7-1.4) ^f^ | 3,039 (33) | 251 (8) | 1.0 (0.9-1.2) | 1.1 (0.9-1.3) ^f^ |
| Alcohol intake |  |  |  |  |  |  |  |  |
| T1 (≤7) | 1,006 (39) | 70 (7) | Reference | Reference ^g^ | 3,538 (32) | 306 (9) | Reference | Reference ^g^ |
| T2 (8-14) | 764 (29) | 46 (6) | 0.9 (0.6-1.2) | 0.9 (0.6-1.2) ^g^ | 2,800 (30) | 213 (8) | 0.9 (0.7-1.0) | 0.9 (0.7-1.0) ^g^ |
| T3 (≥15) | 834 (32) | 50 (6) | 0.9 (0.6-1.2) | 0.9 (0.6-1.2) ^g^ | 3,002 (32) | 202 (7) | 0.8 (0.7-0.9) | 0.8 (0.6-0.9) ^g^ |

Abbreviations: PCa= Prostate cancer, T= Tertile, n= Number, HR= Hazard Ration, aHR= Adjusted Hazard Ratio, CI= Confidence Interval, Ref= Reference, BMI= Body Mass Index, WC= Waist circumference, WHR= Waist-hip ratio, CCI= Charlson’s comorbidity index, MET= Metabolic Equivalents of Task

^a^ Adjusted for the year of index.

^b^ Adjusted for education, Charlson’s Comorbidity Index score, smoking, alcohol, Metabolic Equivalents of Task score, age at index, year of index

^c^ Adjusted for education, BMI, smoking, alcohol, Metabolic Equivalents of Task score, age at diagnosis, year of index

^d^ Adjusted for education, BMI, Charlson’s Comorbidity Index score, alcohol, Metabolic Equivalents of Task score, age at index, year of index

^f^ Adjusted for education, BMI, Charlson’s Comorbidity Index score, alcohol, smoking, age at index, year of index

^g^ Adjusted for education, BMI, Charlson’s Comorbidity Index score, Metabolic Equivalents of Task score, smoking, age at index, year of index

**Supplementary Table 5.** The hazard ratios of type 2 diabetes mellitus associated with first-line treatment and time since diagnosis in the subpopulation of 1,683 men diagnosed with prostate cancer from 1995 through 2013.

|  | **HR (CI 95%)** | **aHR (CI 95%) ^a^** |
| --- | --- | --- |
| **Total follow-up after index** | | |
| Androgen deprivation therapy | 1.1 (0.7-1.7) | 1.1 (0.7-1.8) |
| Conservative treatment | 1.1 (0.7-1.6) | 1.0 (0.7-1.6) |
| Curative treatment | Reference | Reference |
| **0 - 1 year after index date** | | |
| Androgen deprivation therapy | 1.2 (0.5-3.0) | 1.5 (0.6-3.7) |
| Conservative treatment | 1.3 (0.5-3.3) | 1.4 (0.6-3.7) |
| Curative treatment | Reference | Reference |
| **>1 - 5 years after index date** | | |
| Androgen deprivation therapy | 1.1 (0.6-2.1) | 0.9 (0.5-1.8) |
| Conservative treatment | 1.2 (0.7-2.2) | 1.2 (0.6-2.1) |
| Curative treatment | Reference | Reference |
| **>5 - 10 years after index date** | | |
| Androgen deprivation therapy | 1.1 (0.4-2.8) | 1.2 (0.5-3.3) |
| Conservative treatment | 0.7 (0.3-1.7) | 0.5 (0.2-1.4) |
| Curative treatment | Reference | Reference |
| **>10 years after index date** | | |
| Androgen deprivation therapy | 1.4 (0.3-6.4) | 1.3 (0.3-6.7) |
| Conservative treatment | 1.1 (0.3-3.5) | 0.7 (0.2-2.9) |
| Curative treatment | Reference | Reference |

Abbreviations: HR= Hazard ratio, aHR= Adjusted Hazard Ratio, CI= Confidence Interval.

Conservative treatment included active surveillance or watchful waiting. Curative treatment included radical prostatectomy or radiation therapy. ^a^Adjusted for education, smoking, BMI, Charlson’s Comorbidity Index Score, alcohol, Metabolic Equivalents of Task score, age at index, and year of index. There were no significant interactions between androgen deprivation therapy and any covariates (Not shown).
